# Cause Which Retard Dental Progress

**Published:** 1861-11

**Authors:** 


CAUSES WHICH RETARD DENTAL PROGRESS.—*Read before the American Dental Convention, at their meeting in New Haven, Ct., August,* 1861, *by the President,* Dr. ALLEN.—For more than two thousand years dentistry has been gradually advancing, though slowly, yet more and more rapidly as its progress with the march of time has been onward. There may be those who will note the present era as the time when dental surgery reached its ultimatum. So thought many of our predecessors in years gone by; but the future historian will doubtless look back upon the year eighteen hundred and sixty-one as forming only a connecting link between the past and the future, and that a still brighter period in our profession will mark the page of history. The rapid advancement of dental science within the last thirty years is only an index of what is to come. The facilities now afforded for acquiring a thorough dental education through the instrumentality of numerous well qualified private preceptors, together with the dental colleges, dental associations and dental literature, in this country and in Europe, all point with peculiar significance to a brighter future. The march of improvement is still onward; the sun of science continues to shed its genial rays upon our profession, and to develop more perfectly the principles upon which it is based. But still, with all these advantages, and all that has been done to bring dental surgery forward to its present stand-point, much remains yet to be accomplished.

We deem it proper, therefore, to notice some of the causes which retard our onward progress. There are many who never venture out of the old beaten track of their predecessors; they will acquire, perhaps, the same degree of skill, go as far as others have gone, but no farther. They, however, nobly sustain the profession as they find it, and their valuable services have done much to ameliorate the condition of those who were so fortunate as to fall into their hands, and many of their operations will stand as truthful records of their memory, long after they shall have gone to their last home.

If these worthies would venture onward to some unbeaten track, their sphere of usefulness might still be enlarged, for in their reconnoitering they might, perchance, discover some new principle or improved method of accomplishing what is already known, and thus add to the common stock of dental science ; but they decline doing this, and rest upon the laurels already won by others.

Another cause that prevents advancement rests with those who venture on a little way, and then turn back before their point is gained, having reached only the conclusion, that if it were possible to accomplish the object had in view, it would long since have been done by some one else. Suppose Harvey had reasoned thus when he commenced his investigations with reference to the circulation of the blood. If he had, the world might yet have been in darkness upon the subject.

Suppose Columbus, after having made a voyage or two in pursuance of his favorite theory, had failed to discover this continent, (which to others then seemed to exist only in his brain,) and had abandoned his object as hopeless, he would not then have been the means of shaping the destiny of millions of our race, who are now enjoying the benefits of his conceptions, his energy, and perseverance.

If Fulton had listened to the advice of friends, whose minds were not enlightened like his upon the subject of steam-power, he never would have developed that great principle which has revolutionized the commerce of the world. But he saw in his mind's eye the engine working, the steamboat moving, the benefits germinating, which would redound to his honor and to the prosperity of nations yet unborn. That which he saw only within the small compass of his brain, we can now see in the forms of steam ships, steam cars, steam mills, steam factories of such gigantic proportions as to fill the mind with awe, wonder, and surprise.

We might dwell with interest upon the thousands of inventions and improvements that mark the progress of science and civilization, all of which first originated in thought, then dwelt in the secret chambers of the human brain, where they were modeled and perhaps already working in the spirit of man, long before they were clothed with a body, and developed in practical forms.

These examples show us the great importance of vigilance and perseverance also in our profession. Again, it is thought by many that our profession is not sufficiently remunerative to justify much expenditure of time and money, in the preparation and pursuit of dental practice. This feeling begets apathy, which retards our progress. Apathy should not find a lodgment among us, for two reasons : one, because our own prosperity requires constant vigilance ; the other, because the community in which we live claim our best services. But, says one, I have to compete with very incompetent men, who charge but half price for their services, and I must meet them on their own ground. Not so ; this is the wrong ground to occupy ; it is far better to avoid these low competitors, by keeping off their platform. Seek a higher level ; the most effectual means of putting down empiricism is to soar above it, and help others up rather than lower yourself. This position will command a practice both lucrative and honorable, for the public are not so dull of perception as to be unable to discriminate between the true professional man and the one devoid of the essential requisites that characterize a well qualified practitioner, who calls to his aid all those fundamental principles and collateral branches which eminently qualify him for the discharge of his professional duties. To this end, he seeks the aid of physiology, which teaches him the functions of the different parts of the human system ; of anatomy, which unfolds to him the situation, structure and economy of the animal body ; of pathology, which explains to him the nature of diseases, their causes and symptoms ; of therapeutics, which guide him to those curative agents that assist nature in restoring health when attacked by disease. These, together with chemistry, mineralogy, metallurgy, and other minor auxiliaries which are acquired by experience in manipulations, illuminate the mind thus disciplined, and its genial rays reflected upon other minds, dispel the darkness which the empiric leaves in his train ; and thus the public can readily distinguish between the man of science and the mere pretender, and will bestow patronage accordingly.

There is another class of dentists who do but little toward furthering our course, although they are good practitioners, profess strong powers of perception, can see defects still in the dental art that ought to be overcome, but are unwilling to incur the necessary expense and time that may be required to produce the desired results. They are unwilling to prosecute investigations in the dark labyrinth that lies before them, without the hope of reward. We trust the time is not far distant when acceptable provisions will be made by means of which this obstruction will be removed, and all others that tend to impede our progress Let every one feel that he can do something, that there is yet room for him to make his mark, and then much will be accomplished. Bear in mind, that men's acts live after they shall have passed away ; and may some lasting good result from the part we have taken in this life.—*Dental Cosmos.*

